# Post-Legalization Surge and Regional Disparities in Cannabis-Induced Psychiatric Disorders: Multicenter Evidence from Thailand (2021–2024)

**DOI:** 10.1192/j.eurpsy.2026.10873

**Published:** 2026-07-31

**Authors:** S. Joowong, P. Tansupasiri

**Affiliations:** 1 Department of Mental Health, Somdet Chaopraya Institute of Psychiatry, Bangkok; 2 Department of Mental Health, Phisanulok Psychiatric Hospital, Phisanulok, Thailand

## Abstract

**Introduction:**

Following Thailand’s cannabis decriminalization in 2022, a marked rise in psychiatric presentations related to cannabis use has been observed nationwide. While international evidence has linked high-THC cannabis exposure to psychosis and substance-induced disorders, empirical data from Asian settings remain limited. This late-breaking study provides updated national trends and regional variations in cannabis-related psychiatric cases following the policy change.

**Objectives:**

1. To analyze the trend of cannabis-related psychiatric cases in Thailand during the period between pre-policy (2021) and after legalization (2023 and 2024).
2. To examine the association between psychiatric diagnostic codes (F1x.5 and F2x.xx) and cannabis-use history.

**Methods:**

A retrospective multicenter study analyzed data from five psychiatric hospitals representing five major regions of Thailand (North, Northeast, Central, South, and East) between 2021 (pre-policy) and 2024 (post-policy). Demographic and clinical data were extracted from hospital records. Statistical analyses included descriptive statistics, Chi-square tests, and linear regression to assess time trends and gender–diagnosis associations between cannabis- induce psychotic disorders (F1x.5) and schizophrenic spectrum disorders (F2x.xx).

**Results:**

Between 2021–2024, 2,131 cannabis-related psychiatric patients were identified (82.4% male, 17.6% female). Cases increased significantly post-legalization, with an average annual rise of 146.6 cases (β = +146.6, R^2^ = 0.875). Males were more likely to be diagnosed with F1x.5, while females showed higher proportions of F2x.xx (χ^2^ = 122.5, p < 0.001). The proportion of patients with cannabis-use history among F1x.5 diagnoses rose from 62.4% (2021) to 78.3% (2024), indicating a significant upward trend (χ^2^trend = 8.52, p = 0.004),while among F2x.xx patients, it increased from **18.7% to 27.5%** during the same period. Regional analysis revealed higher cannabis-related prevalence in northeastern hospitals (up to 48.3%), whereas southern and northern hospitals showed stronger psychotic disorder patterns.

**Image 1:**
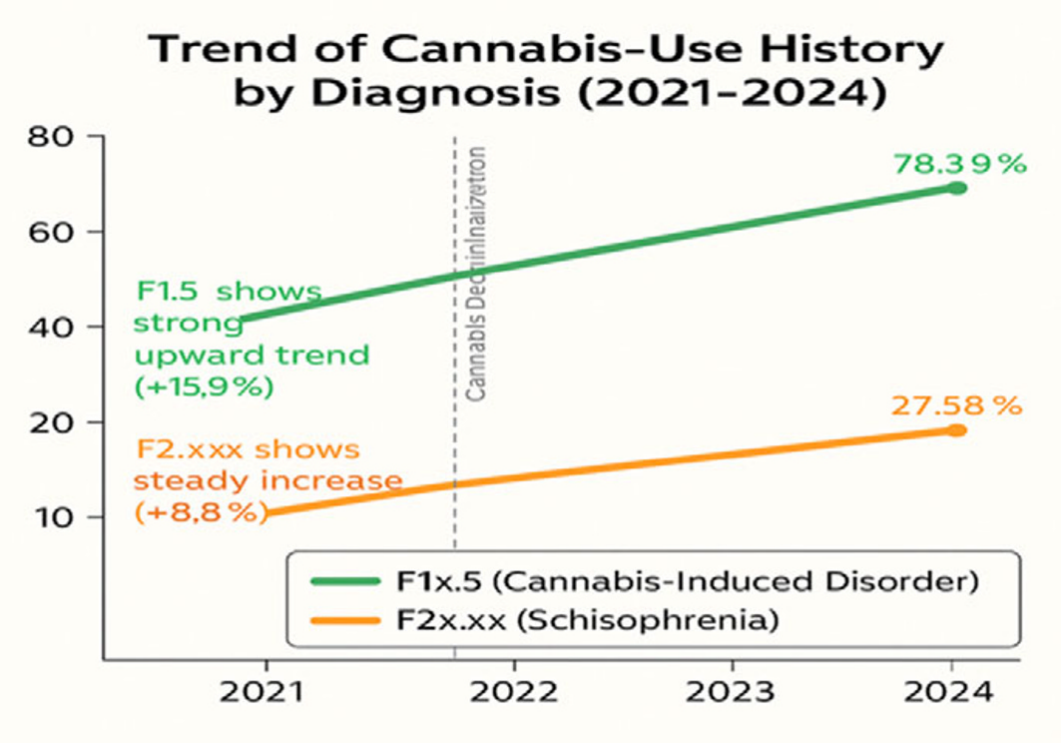
Image 1: Long description.

**Image 2:**
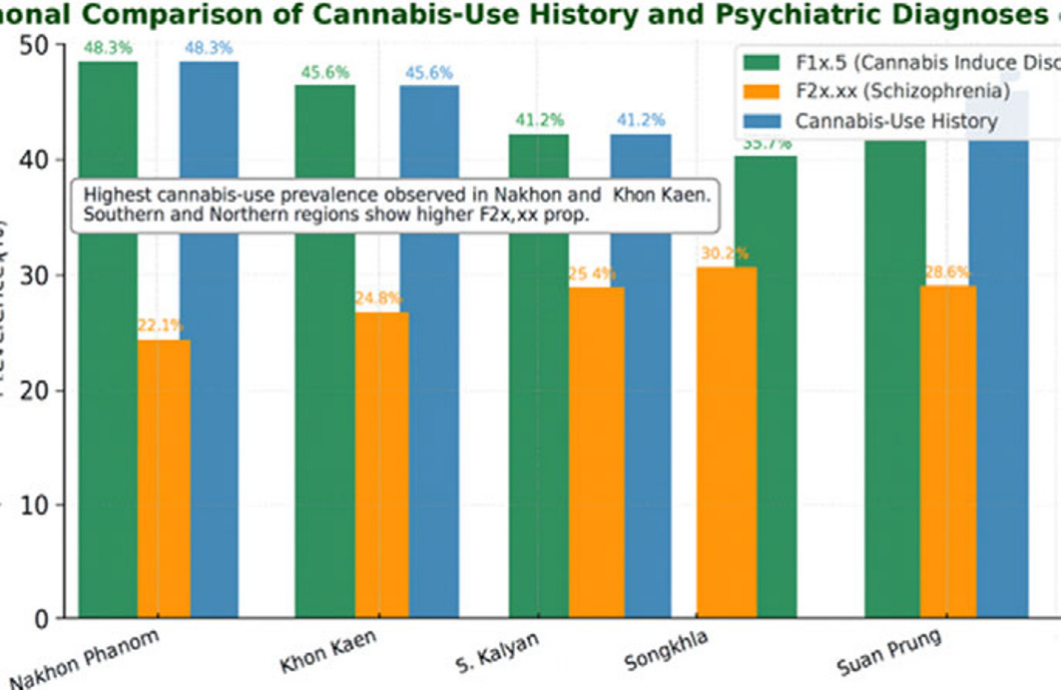
Image 2: Long description.

**Conclusions:**

This study provides one of the first multicenter evidences from Thailand demonstrating a post-legalization rise in cannabis-induced psychiatric morbidity. The results call for innovative, regionally tailored, and gender-sensitive strategies within national mental health surveillance systems.

**Disclosure of Interest:**

None Declared

## Long descriptions

### Image 1: Long description

A line graph titled Trend of Cannabis-Use History by Diagnosis (2021-2024). The Y-axis represents percentage values from 0 to 80, and the X-axis represents years from 2021 to 2024. A legend at the bottom identifies two groups: a green line for F 1 x dot 5 (Cannabis-Induced Disorder) and an orange line for F 2 x dot x x (Schizophrenia).

* The green line (F 1 x dot 5) starts at approximately 40 percent in 2021 and shows a strong upward trend, reaching 78.39 percent in 2024. A text label notes a plus 15.9 percent increase.

* The orange line (F 2 x dot x x) starts at approximately 10 percent in 2021 and shows a steady increase, reaching 27.58 percent in 2024. A text label notes a plus 8.8 percent increase.

A vertical dashed line is positioned between 2021 and 2022 with vertical text that reads Cannabis Decriminalization.

Navigate back to Image 1:.

### Image 2: Long description

The y-axis represents Prevalence in percentage from 0 to 50. The x-axis lists five regions: Nakhon Phanom, Khon Kaen, S. Kalyan, Songkhla, and Suan Prung. A legend in the top right identifies three categories: F 1 x dot 5 (Cannabis Induce Disc) in green, F 2 x dot x x (Schizophrenia) in orange, and Cannabis-Use History in blue.

Data clusters from left to right:

* Nakhon Phanom: Green and blue bars both at 48.3 percent; orange bar at 22.1 percent.

* Khon Kaen: Green and blue bars both at 45.6 percent; orange bar at 24.8 percent.

* S. Kalyan: Green and blue bars both at 41.2 percent; orange bar at 25.4 percent.

* Songkhla: Green and blue bars both at 35.7 percent; orange bar at 30.2 percent.

* Suan Prung: Green and blue bars both at 41.2 percent; orange bar at 28.6 percent.

An annotation box states: Highest cannabis-use prevalence observed in Nakhon and Khon Kaen. Southern and Northern regions show higher F 2 x, x x prop.

Navigate back to Image 2:.

